# Post-Thrombectomy Subarachnoid Hemorrhage: Incidence, Predictors, Clinical Relevance, and Effect Modulators

**DOI:** 10.3390/diagnostics14171856

**Published:** 2024-08-25

**Authors:** Huanwen Chen, Marco Colasurdo, Mihir Khunte, Ajay Malhotra, Dheeraj Gandhi

**Affiliations:** 1 National Institute of Neurological Disorders and Stroke, National Institutes of Health, Bethesda, MD 20892, USA; 2 Department of Neurology, MedStar Georgetown University Hospital, Washington, DC 20007, USA; 3 Department of Interventional Radiology, Oregon Health & Science University, Portland, OR 97239, USA; 4 Warren Alpert Medical School, Brown University, Providence, RI 02903, USA; mihir_khunte@brown.edu; 5 Department of Radiology, Yale New Haven Hospital, New Haven, CT 06510, USA; ajay.malhotra@yale.edu; 6 Departments of Radiology, Neurosurgery, and Neurology, University of Maryland Medical Center, Baltimore, MD 21201, USA

**Keywords:** thrombectomy, stroke, subarachnoid hemorrhage, intracranial hemorrhage, endovascular, medium, distal

## Abstract

Background: Subarachnoid hemorrhage (SAH) following endovascular thrombectomy (EVT) is a poorly understood phenomenon, and whether it is associated with clinical detriment is unclear. Methods: This was an explorative analysis of a national database of real-world hospitalizations in the United States. Patients who underwent EVT were included. Patients were divided into SAH and non-SAH groups, and hospitalization outcomes were compared using multivariable logistic regression models. Regression models were also used to identify significant predictors for post-EVT SAH, and significant modulators of SAH’s association with hospitalization outcomes were also assessed. Results: A total of 99,219 EVT patients were identified; 6174 (6.2%) had SAH. Overall, SAH was independently associated with increased odds of in-hospital mortality (21.5% vs. 10.6%, adjusted OR 2.53 [95%CI 2.23–2.87], *p* < 0.001) and lower odds of routine discharge to home with self-care (18.2% vs. 28.0%, aOR 0.58 [95%CI 0.52–0.65], *p* < 0.001). Distal/medium vessel occlusion (DMVO), coagulopathy, angioplasty or stenting, concurrent intraparenchymal hemorrhage (IPH), and female sex were associated with higher odds of SAH. DMVO was associated with particularly heightened risk of death (31.8% vs. 7.9%, aOR 6.99 [95%CI 2.99 to 16.3], *p* < 0.001), which was an effect size significantly larger than other sites of vascular occlusion (interaction *p* > 0.05). Conclusion: SAH is an uncommon but likely clinically detrimental post-EVT complication. DMVO, coagulopathy, angioplasty or stenting, concurrent IPH, and female sex were independently associated with higher odds of post-EVT SAH. SAH associated with DMVO-EVT may be particularly harmful.

## 1. Introduction

Endovascular thrombectomy (EVT) is standard of care for select patients with acute ischemic stroke; however, it is not without risks [1,2]. Intracranial hemorrhage is a dreaded complication, and it has been shown to be independently associated with poor outcomes [3]. While hemorrhagic transformations of the infarct bed are well-studied with clearly defined diagnostic criteria, post-EVT SAH is a less understood phenomenon. Numerous efforts have been made to continue expanding EVT indications to patients with larger infarcts [4,5,6,7,8,9,10], baseline frailty or underlying medical or neurological comorbidities [11,12,13,14,15,16], presenting in later time windows [17,18,19], and having more distal occlusions [20,21,22,23,24,25,26,27,28,29,30]. Thus, a better understanding of how hemorrhagic complications impact patient outcomes is needed, particularly SAH.

For EVT patients, SAH can occur via various mechanisms, such as vessel wall perforation during endovascular device navigation and manipulation, endothelial irritation during clot retrieval, as well as subarachnoid extension of intraparenchymal blood due to hemorrhagic transformation of ischemic infarct [31]. The presence of SAH may irritate the cerebral cortex and intracranial vasculature, which could increase the risk of seizures, cerebral vasospasm, and delayed hydrocephalus in severe cases [32,33]. Thus, a better understanding of post-EVT SAH is important for optimizing risk–benefit assessments during pre- and intra-operative EVT decision making. Based on prior clinical trial data, post-EVT SAH occurs in approximately 5% of patients with large vessel occlusions, and increased risk has been observed with multiple-pass EVT and distal or medium vessel occlusions (DMVOs). To date, investigations on the impact of post-EVT SAH on clinical outcomes is have yielded mixed results, with some reports suggesting that SAH is an independent risk factor for poor outcomes [31,34], and others suggesting that SAHs may be clinically benign [35,36,37]. Importantly, the current literature is largely based on data from clinical trials and academic medical centers with high procedural volumes, which may not be reflective of routine clinical practice [38]. To date, there have been scarce real-world data on the incidence and clinical importance of post-EVT SAH.

In this explorative analysis of a nationwide database of hospitalizations across community hospitals in the United States, we sought to analyze real-world data on post-EVT SAH to identify its predictors, its impact on outcomes, and factors that modify its clinical relevance.

## 2. Methods

This was a retrospective cohort study of the 2016 to 2021 Nationwide Readmissions Database (NRD), a part of the Healthcare Cost and Utilization Project (HCUP). The NRD is an administrative database that provides stratified real-world discharge information in the United States from community hospitals across 38 states.

Adult patients with acute ischemic stroke who underwent EVT were included. Protocols for patient selection and EVT procedures were based on local institution and provider preferences. To ensure that included patients did not have chronic infarction as a premorbid diagnosis, we excluded patients with missing NIH stroke scale (NIHSS) information. Patients with missing discharge information were excluded.

The primary exposure of interest was SAH, and patients were divided into SAH and non-SAH groups. Patient demographics (age, sex), NIHSS, stroke location, and concomitant intravenous thrombolysis were captured. Stroke location was categorized into anterior versus posterior, as well as by vessel size—large (internal carotid, vertebral, or basilar artery), middle cerebral artery (MCA) tree, and DMVO (anterior and posterior cerebral arteries). The decision to combine all occlusions of the MCA tree into one group was due to limitations of the ICD-10 coding system, which does not allow specification of the MCA segment (M1, M2, M3, etc.). Stroke risk factors such as atrial fibrillation, hypertension, hyperlipidemia, diabetes, smoking history, and congestive heart failure were also extracted, as were anticoagulant use, antiplatelet use, underlying coagulopathy, and concurrent intraparenchymal hemorrhage (IPH). Elixhauser comorbidity index was calculated for each patient to estimate overall medical comorbidity burden [39].

The primary endpoints of this study were rates of in-hospital mortality and routine hospital discharge to home with self-care, which is a surrogate marker for excellent neurological outcomes [26,40]. Variables used for this study were identified using International Classification of Diseases, 10th edition (ICD-10) codes; all codes used for this study are listed in Supplementary Table S1.

### Statistical Methods

The number of patients was calculated using discharge-level weights. Continuous data were expressed as mean ± SD and compared via Student’s *t*-test. Categorial data were represented as percentages and compared with Rao–Scott chi-square tests. Multivariable logistic regression models were used to identify significant predictors for SAH, including a list of variables identified a priori based on prior literature: age, sex, NIHSS, intravenous thrombolysis, concurrent IPH, stroke location, coagulopathy, stroke risk factors (atrial fibrillation/flutter, hypertension, hyperlipidemia, diabetes, congestive heart failure, smoking), antithrombotic medication use (antiplatelet or anticoagulant), and overall comorbidity burden as measured by Elixhauser comorbidity index [39]. Additional interaction analyses were performed to identify significant effect modulators of SAH’s association with discharge outcomes. *p*-values less than 0.05 were deemed statistically significant. All statistical analyses were performed using R, Version 3.6.2.

## 3. Results

### 3.1. Patient Characteristics

A total of 99,219 AIS patients who underwent endovascular thrombectomy with available NIH stroke scale information were identified; 6174 (6.2%) had SAH. Overall, SAH patients were more likely to be female and to have underlying coagulopathy. Furthermore, SAH patients were more likely to have undergone angioplasty or stenting procedures in addition to EVT, and they were more likely to have suffered concurrent intraparenchymal hemorrhage. Full details regarding patient characteristics are presented in Table 1 and Supplementary Table S2.

### 3.2. Impact of SAH on Patient Outcomes

**Figure 1:**
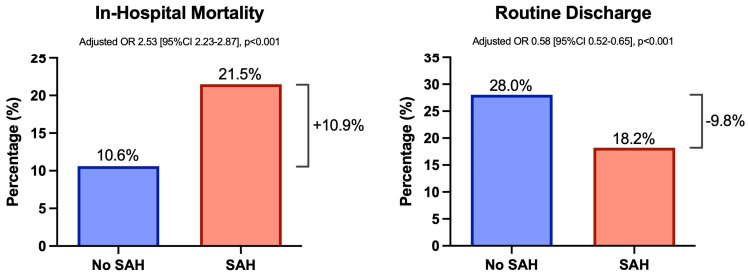
Hospitalization outcomes of patients with or without subarachnoid hemorrhage following endovascular thrombectomy for acute ischemic stroke. Routine discharge was defined as discharge to home to self-care. Multivariable adjustments were made for patient age, sex, site of vascular occlusion, stroke severity, stroke risk factors, antithrombotic medication use, and medical comorbidities. *p*-values less than 0.05 were deemed statistically significant.

SAH was significantly associated with increased rate and odds of in-hospital mortality (21.5% vs. 10.6%, adjusted OR 2.53 [95%CI 2.23–2.87], *p* < 0.001; Figure 1) as well as lower rate and odds of routine discharge (18.2% vs. 28.0%, adjusted OR 0.58 [95%CI 0.52–0.65], *p* < 0.001; Figure 1). In a sensitivity analyses, isolated SAH remained statistically significantly associated with lower odds of routine discharge (adjusted OR 0.64 [95%CI 0.57 to 0.73], *p* < 0.001) and higher odds of death (adjusted OR 2.11 [95%CI 1.83 to 2.44], *p* < 0.001)

### 3.3. Predictors of SAH Risk

In multivariable regression analysis, several factors were significantly associated with higher odds of SAH, including DMVO, coagulopathy, angioplasty or stenting, concurrent IPH, and female sex (Table 2). Long-term anticoagulant use and posterior circulation occlusion were significantly associated with lower risk of SAH (Table 2). In a sensitivity analysis of isolated SAH, female sex, MCA, DMVO, angioplasty/stenting, coagulopathy, and posterior circulation location remained statistically significantly associated with risk of isolated ICH (all *p* < 0.05).

### 3.4. Modulators of SAH’s Association with Poorer Outcomes

**Figure 2:**
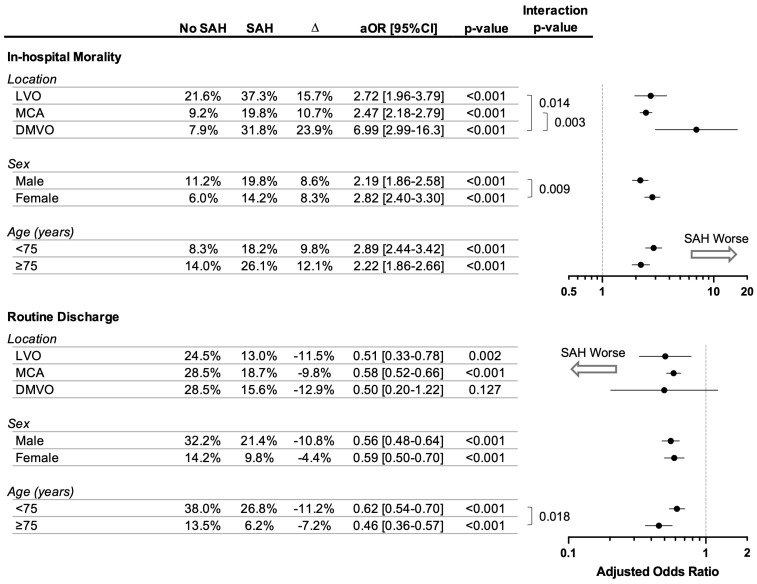
Hospitalization outcomes of patients with or without subarachnoid hemorrhage following endovascular thrombectomy for acute ischemic stroke stratified by patient subgroups. Adjusted odds ratios (aOR) accounted for patient age, sex, site of vascular occlusion, stroke severity, stroke risk factors, antithrombotic medication use, and medical comorbidities. *p*-values less than 0.05 were deemed statistically significant.

Various factors significantly modulated the association of SAH with poorer outcomes. In terms of in-hospital mortality, SAH for patients who underwent EVT in the DMVO location was associated with a particularly heightened risk of death (adjusted OR 6.99 [95%CI 2.99 to 16.3], *p* < 0.001; Figure 2), which was significantly elevated compared to MCA (interaction *p* = 0.003, Figure 2) and proximal LVO locations (interaction *p* = 0.014, Figure 2). Female sex was also significantly associated with an augmentation of increased mortality risk associated with SAH (interaction *p* = 0.009), though the absolute differences in rates of in-hospital death associated with SAH were similar for men and women (8.6% and 8.3%, respectively; Figure 2). Finally, older age (75 years or older) significantly augmented the reduced odds of routine discharge associated with SAH (interaction *p* = 0.018), though the absolute difference was smaller among elderly patients compared to younger patients (7.2% vs. 11.2%, Figure 2). No significant interactions were observed between SAH and all other variables captured in this study in terms of in-hospital mortality or routine discharge (all *p* > 0.05).

## 4. Discussion

In this retrospective analysis of a real-world nationwide database of hospitalizations of the United States, we found that (1) the incidence of SAH among EVT-treated patients was 6.2%, consistent with prior clinical trial data [31], (2) SAH was significantly associated with increased risk of in-hospital mortality and decreased likelihood of good hospitalization outcomes, (3) EVT for DMVOs was associated with a higher risk of SAH, and SAHs associated with DMVO EVTs appeared to carry a particularly elevated mortality risk. Overall, these findings highlight the clinical importance of post-EVT SAHs, particularly for DMVO strokes.

SAH is a known complication of stroke EVT; however, its clinical relevance is not well understood. In addition to the possible extension of post-stroke intraparenchymal hemorrhage into the subarachnoid space, additional factors can contribute to EVT-associated SAH, including endothelial damage during clot retrieval, catheter manipulation, or vessel wall perforation [36]. Unlike intraparenchymal hemorrhagic transformations following ischemic strokes, post-stroke and EVT SAH does not have a universally adopted grading system, and the clinical impact of SAH can vary greatly. While some prior reports have suggested that SAH may be associated with worse outcomes [31,34], others have presented the contrary, suggesting that EVT-associated SAH may be largely clinically benign [35,36,37]. These discrepancies may have stemmed from the small number of patients who experience post-EVT SAH. In this large-scale analysis of 99,219 patients from a real-world dataset, results suggest that SAH is likely associated with net clinical harm. This finding calls for future efforts to better characterize post-EVT SAHs, and to investigate optimal strategies to prevent and manage this poorly understood phenomenon.

Our study also revealed that EVT for DMVO is particularly prone to post-procedure SAH, which corroborates numerous prior reports [26,28,31]. Our study also reports a novel finding that SAHs associated with DMVO EVTs may be more harmful than other EVT-related SAHs in terms of mortality risk. The reasons underlying these findings may be two-fold. First, DMVO strokes are clinically milder. With a smaller treatment target (relief of a milder neurological deficit), patient outcomes may be more sensitive to procedural risks. Second, currently available EVT devices are generally designed for use in large vessels and not optimized for DMVOs, which may have increased the odds of procedural harm [41]. Together, these findings call for careful cost–benefit analyses when considering EVT for DMVO strokes, as well as new thrombectomy devices more tailored for DMVO use. Our study also found that sex and age may modulate the clinical harm of SAH; future work is needed to further explore whether women and elderly patients may warrant additional consideration during EVT decision making and peri-procedural care [16,42].

Our study has several limitations. First, this was a retrospective analysis of an administrative database, and many relevant clinical variables are not available, such as degree of revascularization, grading of intraparenchymal hemorrhagic transformations, proceduralist experience, patient selection protocols, and hospital EVT volume. As such, our study results may have been confounded by these variables that were not available to us. Second, our study outcomes were limited to discharge destinations, and neurological outcomes are available in the database. However, routine discharge is a widely reported and validated surrogate marker for excellent neurological outcomes [26,40], and discharge to home is also a commonly used endpoint [43,44]. Thus, our findings likely remain robust in detecting short-term outcomes as well as hospital death. Third, while we were able to identify posterior and anterior circulation artery occlusions as DMVO, the NRD does not report the location of occlusions within the middle cerebral artery tree. Thus, whether results in our study regarding DMVOs apply to distal MCA (e.g., M3 or M4) occlusions requires confirmation with future studies. Finally, the method with which SAHs were diagnosed was not reported. Although the overall rate of SAH reported in our study (6.2%) is consistent with prior EVT trial data, it is possible that there is under-ascertainment of SAH in real-world practice.

## 5. Conclusions

SAH is an uncommon but likely clinically detrimental complication following EVT. DMVO, coagulopathy, angioplasty or stenting, concurrent IPH, and female sex were independently associated with higher odds of post-EVT SAH, and SAH associated with DMVO-EVT appeared particularly harmful.

## Figures and Tables

**Table 1:** Patient characteristics.

	Total Cohort	No SAH	SAH	
Characteristic—% (n) or Mean (SD)	N = 99,219 (100%)	N = 93,045 (93.8%)	N = 6174 (6.2%)	SMD
Female sex	51.1% (50,657)	50.7% (47,191)	56.1% (3466)	0.109
Age (years)	69.1 (14.8)	69.1 (14.8)	69.6 (14.4)	0.032
NIH Stroke Scale	15.0 (7.8)	15.0 (7.8)	15.1 (7.9)	0.018
Posterior circulation	7.6% (7551)	7.8% (7295)	4.2% (257)	0.185
Site of occlusion				
Large vessel (ICA, BA, VA)	11.7% (11,619)	12.0% (11,119)	8.1% (500)	0.141
Middle cerebral artery (M1, M2, M3 etc.)	85.8% (85,167)	85.6% (79,616)	89.9% (5552)	0.145
Distal/medium vessel (ACA, PCA)	2.5% (2433)	2.5% (2311)	2.0% (122)	0.037
Non-embolic etiology	62.3% (61,822)	62.3% (57,993)	62.0% (3829)	0.006
Bleeding risk factors				
Intravenous tPA use	33.9% (33,666)	33.9% (31,566)	34.0% (2100)	0.002
Anticoagulant use	17.7% (17,580)	17.8% (16,562)	16.5% (1018)	0.035
Antiplatelet use	27.1% (26,930)	27.2% (25,338)	25.8% (1592)	0.033
Coagulopathy	10.4% (10,358)	10.1% (9426)	15.1% (932)	0.139
Stroke risk factors				
Atrial fibrillation or flutter	41.3% (41,008)	41.3% (38,468)	41.1% (2540)	0.004
Hypertension	82.5% (81,820)	82.6% (76,817)	81.0% (5002)	0.039
Smoking history	18.4% (18,258)	18.5% (17,251)	16.3% (1006)	0.061
Hyperlipidemia	58.7% (58,284)	58.9% (54,786)	56.7% (3498)	0.045
Diabetes	11.9% (11,815)	12.0% (11,151)	10.7% (663)	0.040
Diabetes (complicated)	17.5% (17,404)	17.5% (16,288)	18.1% (1117)	0.015
Chronic heart failure	26.8% (26,570)	26.7% (24,853)	27.8% (1716)	0.024
Angioplasty or stenting	10.3% (10,180)	10.1% (9404)	12.6% (776)	0.074
Concurrent intraparenchymal hemorrhage	17.5% (17,366)	17.2% (15,958)	22.8% (1408)	0.135

**Table 2:** Predictors of SAH risk.

Predictor	Adjusted OR [95%CI]	*p*-Value
Higher risk		
DMVO *	1.50 [1.07–2.09]	0.017
Coagulopathy	1.47 [1.33–1.62]	<0.001
Angioplasty	1.44 [1.22–1.70]	<0.001
Concurrent intraparenchymal hemorrhage	1.39 [1.26–1.55]	<0.001
MCA *	1.28 [1.08–1.52]	0.004
Female sex	1.27 [1.17–1.37]	<0.001
No change in risk		
tPA	1.03 [0.94–1.12]	0.55
Non-embolic etiology	1.02 [0.94–1.10]	0.65
Age	1.01 [0.98–1.05]	0.46
NIHSS	0.99 [0.93–1.05]	0.68
Antiplatelet use	0.98 [0.91–1.05]	0.54
Lower risk		
Anticoagulant use	0.89 [0.80–1.00]	0.041
Posterior cirrculation	0.61 [0.48–0.77]	<0.001

*: Compared to ICA or VB.

## Data Availability

Restrictions apply to the availability of these data. Data were obtained from the Healthcare Cost and Utilization Project (HCUP) and are available for purchase at https://hcup-us.ahrq.gov with the permission of HCUP.

## Supplementary Materials

The following supporting information can be downloaded at: https://www.mdpi.com/article/10.3390/diagnostics14171856/s1. Table S1: ICD-10 codes used for this study, Table S2: Other comorbidities
