# Supplementary material for: Post-Thrombectomy Subarachnoid Hemorrhage: Incidence, Predictors, Clinical Relevance, and Effect Modulators
Source: Diagnostics (Basel). 2024 Aug 25;14(17):1856. doi: 10.3390/diagnostics14171856 (PMC11394096; doi:10.3390/diagnostics14171856)
Supplement: Supplementary file 1 [file diagnostics-14-01856-s001.zip › diagnostics-3152047-supplementary.pdf]

Table S1: ICD-10 codes used for this study

	**ICD-10**
**Inclusion**	
Stroke	I63
Endovascular thrombectomy	03CG, 03CH, 03CJ, 03CK, 03CL, 03CP, 03CQ
**Outcome**	
Subarachnoid hemorrhage	I60

	ICD-10 codes
Hyperlipidemia	*E78.00, E78.01, E78.1, E78.2, E78.3, E78.41, E78.49, E78.5*
Smoking	*Z72.0, Z87.891, F17.210*
Hypertension	*I10.x*
Uncomplicated diabetes	*E10.0, E10.1, E10.9, E11.0, E11.1, E11.9, E12.0, E12.1, E12.9, E13.0, E13.1, E13.9, E14.0, E14.1, E14.9*
Complicated Diabetes	*E10.2-E10.8, E11.2-E11.8, E12.2- E12.8, E13.2-E13.8, E14.2-E14.8*
Smoking	*F17.x, Z72.0, Z87.891*
Drug abuse	*F11.x-F16.x, F18.x, F19.x, Z71.5. Z72.2*
Coagulopathy	*D65-D68.x, D69.1, D69.3-D69.6*
Chronic heart failure	*I09.9, I11.0, I13.0, I13.2, I25.5, I42.0, 142.5-I42.9, I43.x, I50.x, P29.0*
NIH Stroke Scale	*R297*
Atrial Fibrillation or Flutter	*I48.0, I481.1, I48.19, I48.20, I48.21, I48.3, I48.4, I48.91, I48.92*
Intracerebral hemorrhage	*I61.x*
tPA in a different facility within the last 24 hours	*Z92.82, 3E03317*

Table S2: Other comorbidities

Comorbidity - % (n)	Total Cohort<br>N = 99,219 (100%)	No SAH<br>N = 93,045 (93.8%)	SAH<br>N = 6,174 (6.2%)	SMD
Valvular disease	13.4% (13,251)	13.3% (12,403)	13.7% (848)	0.012
Pulmonary hypertension	7.6% (7,539)	7.5% (7,015)	8.5% (524)	0.034
Peripheral vascular disease	10.9% (10,828)	10.8% (10,067)	12.3% (761)	0.046
Pulmonary disease	16.1% (16,007)	16.1% (14,966)	16.9% (1,041)	0.021
Hypothyroidism	14.2% (14,094)	14.2% (13,195)	14.6% (900)	0.011
Renal disease	15.6% (15,472)	15.5% (14,435)	16.8% (1,037)	0.034
**Liver disease**	**2.3% (2,233)**	**2.2% (2,042)**	**3.1% (192)**	**0.052***
Peptic ulcer disease	0.8% (763)	0.8% (716)	0.8% (46)	-0.002
HIV	0.2% (222)	0.2% (212)	0.2% (11)	-0.014
Lymphoma	0.5% (508)	0.5% (467)	0.7% (41)	0.020
**Metastatic cancer**	**2.4% (2,354)**	**2.3% (2,130)**	**3.6% (223)**	**0.071***
Solid tumor	2.5% (2,502)	2.5% (2,314)	3.0% (188)	0.032
Rheumatic disease	2.6% (2,614)	2.6% (2,417)	3.2% (197)	0.034
**Obesity**	**16.5% (16,409)**	**16.7% (15,546)**	**14.0% (864)**	**0.078***
**Weight loss**	**6.2% (6,104)**	**6.0% (5,591)**	**8.3% (513)**	**0.083***
Blood loss	0.6% (613)	0.6% (572)	0.7% (41)	0.005
Anemia	4.2% (4,172)	4.2% (3,888)	4.6% (284)	0.020
Alcohol use	5.4% (5,351)	5.4% (5,025)	5.3% (325)	-0.006
Drug use	4.6% (4,516)	4.5% (4,233)	4.6% (283)	0.002
Psychoses	0.9% (859)	0.9% (816)	0.7% (43)	-0.021
Depression	12.5% (12,389)	12.5% (11,619)	12.5% (770)	0.000
Myocardial infarction	11.6% (11,520)	11.6% (10,782)	12.0% (738)	0.012
Dementia	6.2% (6,108)	6.2% (5,728)	6.2% (380)	0.000
Non-stroke paralysis	0.7% (655)	0.7% (620)	0.6% (35)	-0.014
**Non-AF arrhythmia**	**12.6% (12,453)**	**12.4% (11,535)**	**14.9% (919)**	**0.070***
